# Study of the Activity and Possible Mechanism of Action of a Reversible Inhibitor of Recombinant Human KAT-2: A Promising Lead in Neurodegenerative and Cognitive Disorders

**DOI:** 10.3390/molecules21070856

**Published:** 2016-06-29

**Authors:** Alireza Nematollahi, Guanchen Sun, Gayan S. Jayawickrama, Jane R. Hanrahan, W. Bret Church

**Affiliations:** 1 Group in Biomolecular Structure and Informatics, Faculty of Pharmacy, University of Sydney, Sydney, NSW 2006, Australia; gsun2866@uni.sydney.edu.au (G.S.); gjay8197@uni.sydney.edu.au (G.S.J.); 2 Faculty of Pharmacy, University of Sydney, Sydney, NSW 2006, Australia; jane.hanrahan@sydney.edu.au

**Keywords:** Kynurenine aminotransferase-2, (*S*)-ESBA, PF-04859989, BFF-122, SPR

## Abstract

Abnormal levels of kynurenic acid (KYNA) in the human brain are believed to be connected to several central nervous system (CNS) diseases, therefore compounds which affect the production of this crucial metabolite are of interest in CNS drug development. The majority of KYNA production is accounted for by kynurenine aminotransferase-2 (KAT-2) in the mammalian brain; hence this enzyme is one of the most interesting targets with which to modulate KYNA levels. Recently developed human KAT-2 inhibitors with high potencies are known to irreversibly bind to the enzyme cofactor, pyridoxal-5′-phosphate (PLP), which may lead to severe side effects due to the abundance of PLP-dependent enzymes. In this study, we report a reversible and competitive inhibitor of KAT-2. Its inhibitory activities were examined using HPLC and surface plasmon resonance (SPR) and compare favorably with other recently reported KAT-2 inhibitors. Our inhibitor, NS-1502, demonstrates suitable inhibitory activity, almost 10 times more potent than the known reversible KAT-2, (*S*)-ESBA.

## 1. Introduction

**Figure 1:**
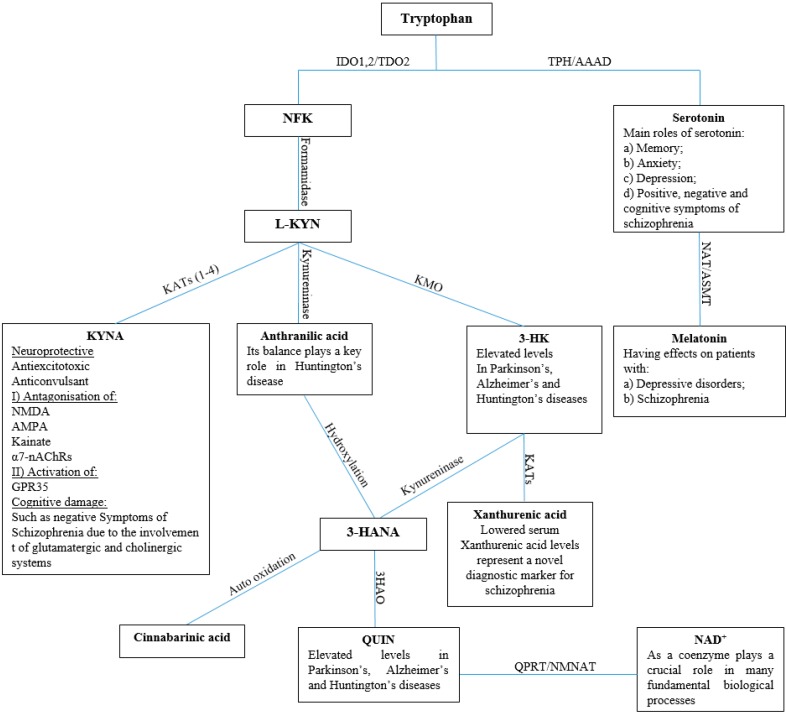
The tryptophan catabolic pathway consists of two routes. One is serotonin biosynthesis, the route which is determined by the amount of tryptophan which can cross the blood-brain barrier (BBB). As illustrated, two enzymes transform tryptophan into serotonin: first tryptophan hydroxylase (TPH) hydroxylates tryptophan into 5-hydroxytryptophan and then in the second step l-aromatic amino acid decarboxylase (AAAD) decarboxylases 5-hydroxytryptophan into serotonin. Serotonin is consequently transformed to an active metabolite, melatonin, via two-steps of enzymatic catabolism. Firstly, serotonin is transformed to *N*-acetylserotonin via acetylation of its amine group by *N*-acetyl transferase (NAT), and then acetylserotonin *O*-methyltransferase (ASMT) catalyzes the subsequent transformation, converting normelatonin to melatonin. The second route is known as the kynurenine pathway, which is the main route of tryptophan metabolism. This pathway is started by the conversion of tryptophan to *N*-formylkynurenine (NFK) by indoleamine 2,3-dioxygenase 1 (IDO1) and tryptophan 2,3-dioxygenase 2 (TDO2). L-KYN is obtained by the metabolism of NFK by formamidase. There are three branches for subsequent L-KYN metabolism. One is in which it is converted irreversibly into KYNA by kynurenine aminotransferases (KATs) isozymes. In the other branches, kynurenine 3-monooxygenase (KMO) and kynureninase transform L-KYN into 3-hydroxykynurenine (3-HK) and anthranilic acid, respectively. After that, anthranilic acid and the 3-HK are transformed by hydroxylation or kynureninase to a mutual product: 3-hydroxyanthranilic acid (3-HANA), and in the second step of the branch the 3-HK acts as a substrate of the KAT isoenzymes, and is catalysed into xanthurenic acid. An auto oxidation reaction converts 3-HANA into cinnabarinic acid; alternatively, 3-HANA is transformed into quinolinic acid (QUIN) by 3-hydroxyanthranilic acid 3,4-dioxygenase (3HAO). In the last steps of this branch, one of the crucial mammalian cofactors, nicotinamide adenine dinucleotide^+^ (NAD^+^), is produced by quinolinate phosphoribosyl transferase (QPRT) and nicotinamide nucleotide adenylyl transferase (NMNAT), respectively.

Tryptophan, an essential amino acid easily obtained from the diet, is able to cross the blood brain barrier (BBB) with the aid of its specialized transporter protein. Within the central nervous system (CNS), it is involved in several important catabolic pathways including those of serotonin and kynurenine [1,2]. The overview of tryptophan catabolism with relationships with CNS diseases [3,4,5,6,7,8,9,10,11] is illustrated in Figure 1.

**Figure 2:**
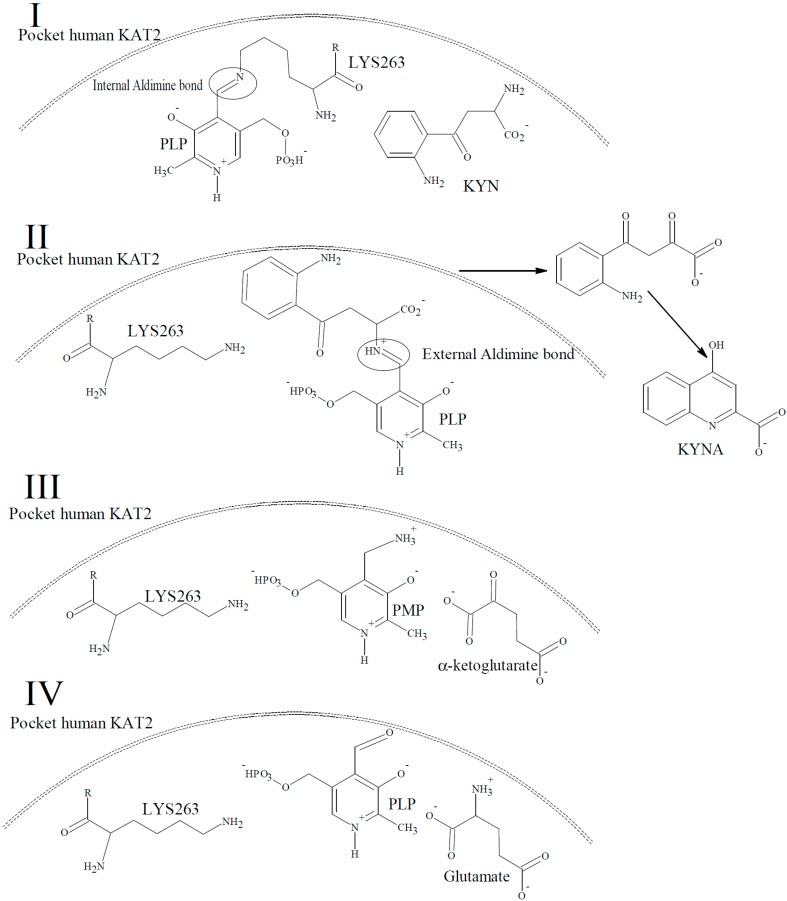
The mechanism of the human KAT-2 catalytic cycle. (**I**) PLP is covalently bound to LYS263 in the reaction pocket of human KAT-2 by an internal aldimine linkage in the native form of the enzyme; (**II**) The α-amino group of KYN in the active site binds to PLP via an external aldimine bond, and breaks the internal aldimine bond. Spontaneously ring closure of the α-keto analogue yields KYNA. (**III**) The α-amino group of the external aldimine bond from the previous step is transferred to the PLP, and of the production of KYNA simultaneously allows the formation of pyridoxamine phosphate (PMP); (**IV**) The regeneration of PLP from PMP is accomplished by converting the co-substrate, α-ketoglutarate, to glutamate by transferring the amino-group of PMP to the α-ketoglutarate, and consequently the PLP-form of the enzyme (I) is regenerated.

The members of the pyridoxal-5′-phosphate (PLP)-dependent enzyme family called kynurenine amino transferases (KATs) act as a catalyst in the irreversible cyclic transamination of kynurenine (KYN) to kynurenic acid (KYNA) in the tryptophan metabolic pathway [12]. The mechanism of catalytic cycle of this reaction is shown in detail using KAT2 as the example in Figure 2.

Studies on the cerebral spinal fluid (CSF) of patients diagnosed with schizophrenia and manic depression revealed a significant increase in KYNA levels [13], which is part of the logic that KYNA plays a key role in modulating cognitive functions [14]. Developing KAT isozymes inhibitors is the main strategy to decrease levels of KYNA in the brain, which is beneficial in managing and treating psychiatric disorders [15]. Among the four KATs, KAT2 is the main isozyme responsible (>70%) for KYNA production in the human brain [16], and therefore the inhibition of KAT2 has become the target of investigations. (*S*)-4-(Ethyl sulfonyl)benzoylalanine ((*S*)-ESBA) is the first known reversible KAT2 inhibitor with a reported IC_50_ at around 1 to 2 mM [17]. The next generation of hKAT2 inhibitors were BFF-122 and the cyclic hydroxamic acid PF-04859989, which were identified as potent and irreversible inhibitors of human KAT II with reported IC_50_s of around 0.1 to 1 μM [18]. The mechanism of action of these inhibitors is by inactivating PLP irreversibly, and hence providing the knock down of KAT2—however irreversible inhibitors are generally not optimal in drug design and development [19]. Moreover, Schwarcz and others have recently shown the superior application of the reversible inhibition by using BFF-816 in preclinical trials [20]. It is noteworthy that the irreversible inhibition of PLP (active form of vitamin B_6_) has a range of potential undesirable and dangerous side effects due the interference with the activity of over 300 PLP-dependent enzymes and proteins [21]. It is not far from the reality to state that nearly every major biological system in the human body could be affected directly or indirectly by the inactivation of PLP which is needed in more than 140 different enzymatic pathways [22]. Carbidopa and benserazide are typical examples of drugs that permanently deactivate PLP by irreversibly binding to it. The usage of carbidopa and benserazide resulted in multiple adverse effects. From studies on carbidopa which are more comprehensive, after the approval of L-dopa/carbidopa as a combination drug by the FDA, the death rate of Parkinson’s disease (PD) patients was observed to increase, and unfortunately this trend has continued uninterrupted for almost 40 years [23]. The KAT-2 structure consists of three domains including the *N*-terminal arm, the large domain, and the C-terminal domain (called the small domain) belonging to fold type I aminotransferases from PLP-dependent enzymes [24]. Therefore, irreversible inhibitors of KAT-2 are likely to have similar issues as carbidopa and benserazide. 

In the current study, our main goal has been to design and develop novel and reversible KAT-2 inhibitors with suitable potency. Furthermore, in order to comprehensively study KAT-2 inhibitors, the inhibitory activities of the designed compound along with several other reported KAT-2 inhibitors were evaluated using our HPLC based inhibition assay. Meanwhile, by means of HPLC and surface plasmon resonance (SPR) techniques, the probable mechanism of action and binding was identified and described in comparison with PF-04859989, a well-established irreversible KAT-2 inhibitor [19].

## 2. Results and Discussion

### Characterization of the Inhibitory Activity and Binding Affinity of 2-(5,6-Dichloro-1,3-dioxo-1,3-dihydro-2H-isoindol-2-yl)-3-phenylpropanoic Acid (NS-1502) on hKAT2

**Figure 3:**
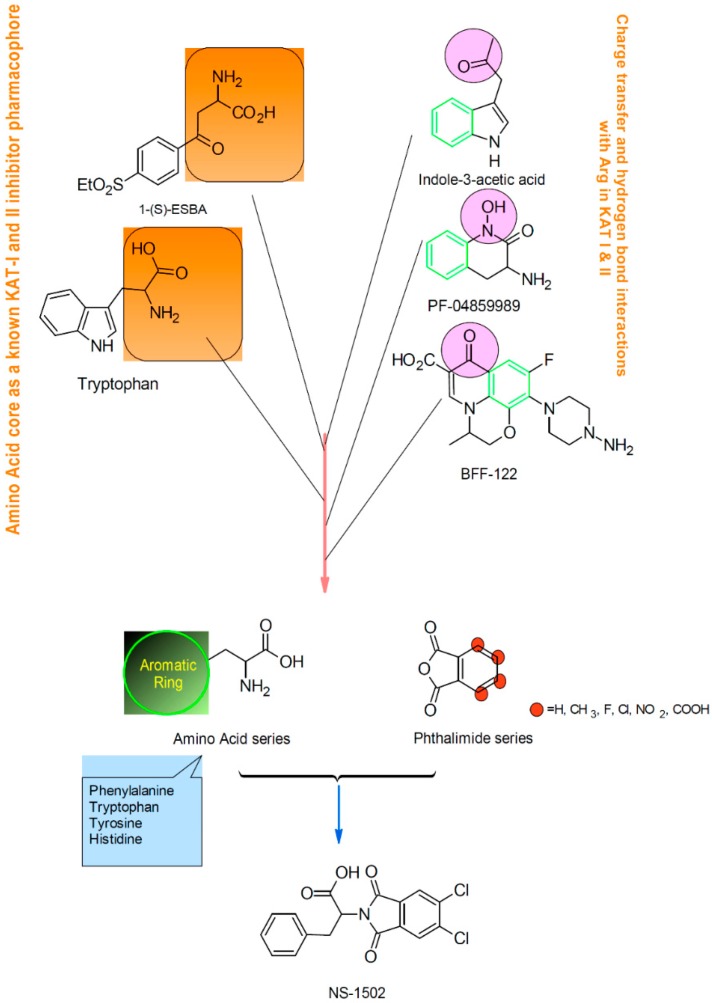
NS-1502 was designed on the basis of existing KATs inhibitors and compounds involved in the kynurenine pathway. The left branch of the design is on the basis of the similarity of (S)-ESBA to KYN, and tryptophan, the right side of the design scheme is based on the pharmacophore of the most potent available KAT-2 inhibitors (BFF-122 and PF-04859989) and general KATs inhibitor (indole-3-acetic acid).

**Figure 4:**
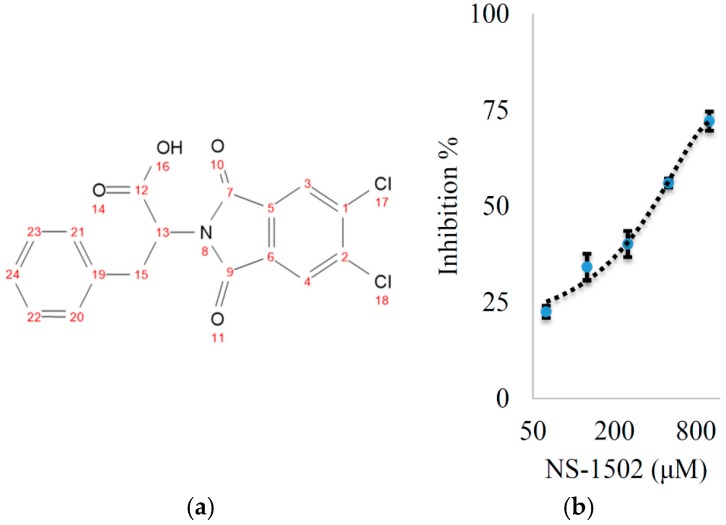
(**a**) Structure of NS-1502 with numbering corresponding to the NMR results; (**b**) Inhibitory activity of NS-1502 in a dose-dependent format.

The design scheme (Figure 3) has two main components. One is based on (*S*)-ESBA, previously designed on the basis of a clear resemblance to the structure of KYN as well as tryptophan (see Figure 1). The second component is based on the development of BFF-122 and PF-04859989, both selective KAT-2 inhibitors, and indole-3-acetic acid. The first step of the pharmacophore design of the general KAT inhibitor was omitting the amine group of BFF-122 and PF-04859989 due to the adverse effects described for the PLP irreversible deactivators. After testing, it was noted that the lipophilic moiety on phthalimide core, led to increased potency, and amino acids with an aromatic ring were considered the most suitable candidates (Figure 3). Finally, the phthalimide with dichloro groups and the amino acid phenyl alanine, NS-1502, was revealed to be a promising inhibitor with using a reversible inhibitory effect on hKAT2 (IC_50_ ≈ 315 μM) (Figure 4).

In comparison with the known and reversible KAT-2 inhibitor, *S*-ESBA, 20% inhibition at 200 μM, NS-1502 displayed a higher potency to inhibit recombinant KAT-2. The calculated IC_50_ values based on the measurement of our protocol were 15–20 μM and 1–3 μM for BFF-122 and PF-04859989, respectively. From this inhibition, NS-1502 fits our main goal of designing a reversible inhibitor that acts without affecting PLP availability, and has increased potency compared to the prior well-established reversible inhibitor (*S*-ESBA). 

**Figure 5:**
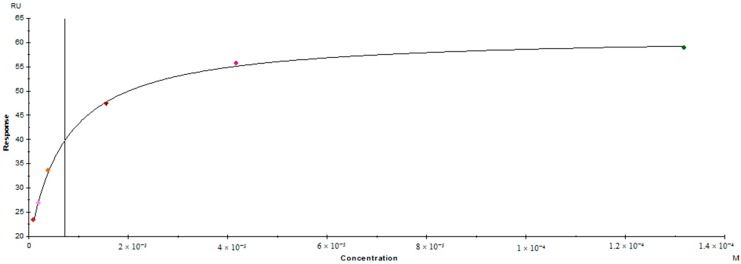
Steady state affinity model obtained of NS-1502 with KAT-2 at 25 °C.

To examine the affinity of the interaction between KAT-2 and NS-1502, we used surface plasmon resonance as a highly sensitive assay that measures changes in the refractive index of the surface of the sensor chip. We were able to achieve sufficient binding levels of KAT-2 onto a CM5 chip via the amine coupling, (20602 RU) to enable quantification of the subsequent analyte interaction with the NS-1502. Solvent corrections were applied to the analysis, and the data demonstrated clear interactions between NS-1502 and KAT-2 in comparison to the referenced flow cell. A steady state affinity model was generated to fit the reference data (Figure 5), showing that NS-1502 binds to KAT-2 with suitable affinity (KD: 7.2 × 10^−6^, chi^2^: 0.34).

Previous studies have demonstrated the reversibility and competitive properties of KAT inhibitors by considering different PLP concentrations on the extent of inhibition in the assay reaction mixture [25,26]. By testing a fixed concentration of inhibitor (500 μM) in the HPLC based inhibition assay with different concentrations of PLP (50, 100, 200 and 500 μM), NS-1502 showed progressively decreased inhibitory activity in response to increased PLP concentrations, while the activities of the well-established irreversible inhibitor, PF-04859989, remained unaffected by the changing PLP concentrations. Our results have confirmed the reversibility of the inhibition of KAT-2 by NS-1502, and also suggested the irreversibly inhibited KAT-2 by PF-04859989 cannot be regenerated by adding extra PLP. Moreover, from experimental results and free binding energy estimation for interactions of PLP with compounds similar to PF-04859989 [27], it is expected that these irreversible inhibitors tend to attack the internal aldimine linkage between PLP and LYS263 in KAT-2 instead of having interaction with free PLP in the reaction environment. Using online prediction tool, the possibility to cross blood-brain barrier (BBB) was measured (http://www.cbligand.org/BBB) based on the threshold and indicates that NS-1502 can probably pass BBB; however, appropriate studies in vivo is necessary.

## 3. Materials and Methods

### 3.1. General Procedures

Commercially available reagents were used without additional purification unless otherwise stated. (*S*)-ESBA, BFF-122 and PF-04859989 were bought from Santa Cruz Biotechnology (Santa Cruz, CA, USA), Axon Medchem (Groningen, The Netherlands) and Sigma (Munich, Germany), respectively. Thin layer chromatography (TLC) was performed on aluminum sheets coated with silica gel (Merck, 60, F254) and examined under UV/VIS light (254 and 365 nm). ^1^H-NMR (400 MHz) and ^13^C-NMR (100 MHz) spectroscopy were obtained using DMSO as NMR solvent on a Gemini 400-MR NMR spectrometer (Varian, Palo Alto, CA, USA). Low and high resolution mass spectroscopy were accomplished by direct infusion electrospray ionization and reported as mass to charge ratio (*m*/*z*) and related intensity (%). Low-resolution ESI-MS was recorded on a TSQ 7000 (LC-MS/MS system, Thermo-Finnigan, San Jose, CA, USA). High resolution ESI-MS was measured on a Apex Ultra Fourier Transform Ion Cyclotron Resonance 7 T Mass Spectrometer (Bruker Daltonics, Bremen, Germany). Surface plasmon resonance (SPR) experiments were performed using a Biacore T200 instrument with a CM5 sensor chip (GE Healthcare Life Sciences, Sydney, Australia), and the data was analyzed using Biacore T200 Control Software and Biacore T200 Evaluation Software. PBST (phosphate buffered saline, pH 7.4 with 0.05% Tween 20) was purchased from Sigma, and SPR buffer (40 mM EDC, 10 mM sulfo-NHS, 10 mM sodium acetate, 1 M ethanolamine HCl, pH 8.5) were purchased from Bio-Rad Laboratories (Gladesville, Australia). Dimethyl sulfoxide (DMSO) was purchased from Thermo Fisher Scientific (Scoresby, Australia).

### 3.2. Inhibitor Synthesis

A solution of 4,5-dichlorophthalic anhydride (5 mmol) and phenylalanine (5 mmol) in glacial acetic acid (7 mL) was stirred and heated under reflux for 6 h. The product of this reaction (Figure 4a, yield 85%) was precipitated by addition of cold water, filtered and dried to afford grams and % yield of 2-(5,6-dichloro-1,3-dioxoisoindolin-2-yl)-3-phenylpropanoic acid (NS-1502). ^1^H-NMR (400 MHz, DMSO-*d*_6_): δ 8.16 (2H, s, H-3,4), 7.15 (5H, m, H-20-24), 5.11 (1H, dd, *J* = 11.53, 4.82 Hz, H-13), 3.48 (1H, dd, *J* = 14.20, 4.82 Hz, H-15a), 3.30 (1H, dd, *J* = 14.20, 11.53 Hz, H-15a); ^13^C-NMR (100 MHz, DMSO-*d*_6_): δ 33.85 (C-15), 53.41 (C-13), 125.68 (C-3,4), 126.59 (C-24), 128.32 (C-20,21), 128.66 (C-22,23), 130.33 (C-5,6), 137.11 (C-19), 138.06 (C-1,2), 165.17 (C-7,9), 169.64 (C-12); HRESIMS: 385.99630 *m*/*z* [C_17_H_11_Cl_2_NO_4_ + Na]^+^ (Calcd. 385.99573).

### 3.3. Protein Preparation

The recombinant KAT-2 protein was expressed and purified in our lab as previously described [28].

### 3.4. Inhibition Studies Using Recombinant Human KAT2

The extent of the conversion of KYN to KYNA was assessed in our inhibition assay by incubating 0.5 μg of KAT-2 at 37 °C for 10 min in a total volume of 50 μL of a reaction mixture containing 50 μM PLP, 5 mM α-ketoglutarate, 5 mM l-KYN in PBS, pH 7.4, with the inhibitor being studied (1–2000 μM). The reaction was terminated by adding an equal volume of formic acid (0.8 M). The KYNA produced was analyzed using HPLC with UV detection at a wavelength of 330 nm using a C18 reverse-phase column using 50% (*v*/*v*) methanol and 50% (*v*/*v*) water as the mobile phase.

### 3.5. Surface Plasmon Resonance Binding Assay

NS-1502 in the prepared solution was passed over the purified expressed his-tagged KAT-2 bound to a CM5 chip. The binding affinity of NS-1502 to KAT-2 was determined by the system recording changes in the refractive index of the surface of the CM5 sensor chip that occurred when the NS-1502 samples interacted.

Prior to the measurements to bind ligand on the chip, solutions were filter sterilized for 10 min at 25 °C and the purified hKAT2 was diluted to a final concentration of 320 µg/mL in sodium acetate buffer (pH 4.5). The flow rate was set to 10 µL/min and the temperature set to 25 °C. The surface of two flow cells was activated using a 1:1 mixture of 100 mM EDC and 100 mM Sulfo-NHS. KAT-2 was subsequently injected for 7 min in flow cell 2. Flow cell 1 was a control immobilization using running buffer (PBST) as a reference. Both flow cells were then deactivated using 1 M ethanolamine. To measure the affinity of NS-1502, different concentrations of the inhibitor samples were prepared (ranging from 1 to 130 µM) in running buffer (PBST with 5% DMSO) immediately before starting the measurements. The flow rate was set to 30 µL/min and all measurements were performed at 25 °C using the multicycle kinetics mode. For the interaction of NS-1502 with the bound KAT-2, the contact time was set to 90 s and the dissociation time to 300 s with an extra 50% DMSO wash performed after each injection. Solvent correction was performed before and after the series of NS-1502 injections due to the use of DMSO in sample preparation and the running buffer. 

## 4. Conclusions

In summary, we have developed a new reversible KAT-2 inhibitor with potency in a similar range to the currently reported irreversible inhibitors. The indication that NS-1502 probably can cross the BBB identifies this molecule as a very promising candidate for lead optimization. Furthermore, we expect our general ideas and the assay and analysis techniques developed may become useful methods for screening and characterizing other novel leads to inhibit this essential enzyme involved in many cognitive and neurodegenerative disorders. Future in vivo studies will be required to further consider the detail required of the effects of this compound on the other enzymes involved in CNS diseases with cognition and psychosis.
